# Complete Necrosis of Eight Teeth from a Blow

**Published:** 1871-08

**Authors:** W. Geo. Beers


### ARTICLE VIII.

### *Complete Necrosis of Eight Teeth from a Blow.*

By W. Geo. Beers, L. D. S.,

A little over two years ago, a vigorous young friend of mine, aged twenty, received a severe blow directly under the nose, while in the act of shouting to a fellow player during a game of Lacrosse. The upper lip was considerably cut, and the crown of the left superior central incisor was broken transversely with the pulp chamber, leaving the pulp hanging partly out of the cavity. As he insisted upon continuing the game, I improvised a barbed brooch by jagging the sides of a common pin with a penknife, and then crooking its point, and succeeded in extirpating the pulp entire, with little or no pain. I observed, as something unusual, that the projecting portion was not at all sensitive to the prick of the pin, while that situated higher up in the cavity was extremely so, and actually reflected a shooting pain along the branch of the nerve. The adjoining teeth did not present any appreciable indications of serious results from the blow, and only the remaining portion of the broken tooth and the adjacent lateral incisor were at all tender to the tap of an excavator, while there was no inflammation of the gums and the cut lip occasioned more pain than the broken tooth.

The second evening after the accident there was a slight tumefaction over the fractured tooth, and both it and the lateral were more tender at the root than the previous day. I applied two leeches to the gums, and the third day all pain had completely subsided, and at the expiration of three weeks, I ventured to insert a pivot tooth to replace the one broken; taking the precaution,—which I considered to be necessary—to prepare the root one day and pivot a few days afterwards. Up to the last day I saw him, he had never had a moment's pain or soreness in this root, but had intermittent pains of a few days duration in the adjoining lateral for over two months, feeling, as he described it, like the knitting of a fractured bone.

About a year ago he came to have his teeth examined before going to Chicago, where he had decided to settle with a newly married wife. I found the pivoted root sound; the troublesome lateral perfectly comfortable. I filled two large cavities with gold in the first inferior molars—the only fillings he had ever had—and after carefully examining the front teeth, I felt justified in prophecying for them a long lease of life. Last January he returned to Montreal, and judge of my surprise to see him come into my house nearly edentulous. The pivot tooth and root, and the adjoining lateral, perfectly free from caries, were easily moved about with his tongue and lip; the cuspids were both loose; the right lateral and the two right bicuspids had actually dropped out of the alveoli. The superior molars and all the teeth of the inferior maxillary were perfectly firm and sound, otherwise I might have suspected mercury, though there were none of the special indications of mercuralization then present; and my patient assured me on his word of honor—which was perfectly reliable—that he had never to his knowledge taken a grain of medicine in any shape or form for the last twelve years, and that he had never had any venereal disease. His parents were both hale and hearty Highlanders, and I would not consider his constitution to be one as easily irritated as some systems are.

The gum and alveolar processes had receded from the necks of the teeth, but it was very clear that the cause did not lie there. There was no perceptible discharge of pus, no pain, no tenderness. The pulps were dead, but the teeth had not assumed the dark hue noticeable in many cases of necrosis.

The alveoli had exfoliated where the lateral and bicuspids had dropped out.

I extracted the lateral and two centrals, and found, as I had anticipated, complete necrosis; the fangs denuded of living periosteum, and covered with little grey and green nodules, similar under the microscope, to salivary calculus. The change in the appearance of the cementum from its normal condition was very striking. A section of dentine of the lateral incisor, under the microscope, revealed the calcification of the dentinal fibres.

I must confess to have been sorely puzzled about this case, presenting, as it does, peculiarities seldom observed in the one mouth, and, what seems to me to be, contradictory characteristics in the course of the disease, and being marked by a complete absence of pain, and no perceptible effusion of pus. I would not have been greatly surprised to have found periodontitis and suppuration from the pivoted root, or even the wounded lateral; but I was shocked to witness the complete destruction, without the barest possibility of cure, of eight teeth, seven of which a year ago were free from decay, and presented fair appearance of a long life. Had inflammation of the periosteum supervened from the blow, the usual indications would be expected, but there were comparatively none of the simplest, and absolutely none of the most marked. The very idea I had in extracting the pulp at the time of the accident was to prevent an extension of the inflammation through its reflecting media to the surrounding membrane, and I think the rather remarkably favorable condition of the parts afterwards, may be attributed to this reason, though it is a question with me now if it might not, perhaps, have been better to have extracted the fractured tooth.—*Canada Journal of Dental Science.*

---

